# The crosstalk between primary MSCs and cancer cells in 2D and 3D cultures: potential therapeutic strategies and impact on drug resistance

**DOI:** 10.1093/stcltm/szae077

**Published:** 2024-10-10

**Authors:** Ayesha Rehman, Sameer Kumar Panda, Martina Torsiello, Martina Marigliano, Camilla Carmela Tufano, Aditya Nigam, Zahida Parveen, Gianpaolo Papaccio, Marcella La Noce

**Affiliations:** Department of Experimental Medicine, University of Campania “L. Vanvitelli” via L. Armanni, 5-80138 Naples, Italy; Department of Experimental Medicine, University of Campania “L. Vanvitelli” via L. Armanni, 5-80138 Naples, Italy; Department of Experimental Medicine, University of Campania “L. Vanvitelli” via L. Armanni, 5-80138 Naples, Italy; Department of Medicine, Surgery and Dentistry “Scuola Medica Salernitana,” Via Salvador Allende, 43, Baronissi, Salerno, Italy; Department of Experimental Medicine, University of Campania “L. Vanvitelli” via L. Armanni, 5-80138 Naples, Italy; Department of Experimental Medicine, University of Campania “L. Vanvitelli” via L. Armanni, 5-80138 Naples, Italy; Department of Experimental Medicine, University of Campania “L. Vanvitelli” via L. Armanni, 5-80138 Naples, Italy; Department of Experimental Medicine, University of Campania “L. Vanvitelli” via L. Armanni, 5-80138 Naples, Italy; Department of Experimental Medicine, University of Campania “L. Vanvitelli” via L. Armanni, 5-80138 Naples, Italy

**Keywords:** MSCs, adipose stem cells, cancer stem cells, microenvironment, stem cell-microenvironment interaction

## Abstract

The tumor microenvironment (TME) significantly influences cancer progression, and mesenchymal stem cells (MSCs) play a crucial role in interacting with tumor cells via paracrine signaling, affecting behaviors such as proliferation, migration, and epithelial-mesenchymal transition. While conventional 2D culture models have provided valuable insights, they cannot fully replicate the complexity and diversity of the TME. Therefore, developing 3D culture systems that better mimic in vivo conditions is essential. This review delves into the heterogeneous nature of the TME, spotlighting MSC-tumor cellular signaling and advancements in 3D culture technologies. Utilizing MSCs in cancer therapy presents opportunities to enhance treatment effectiveness and overcome resistance mechanisms. Understanding MSC interactions within the TME and leveraging 3D culture models can advance novel cancer therapies and improve clinical outcomes. Additionally, this review underscores the therapeutic potential of engineered MSCs, emphasizing their role in targeted anti-cancer treatments.

## Graphical Abstract

**Graphical Abstract:**
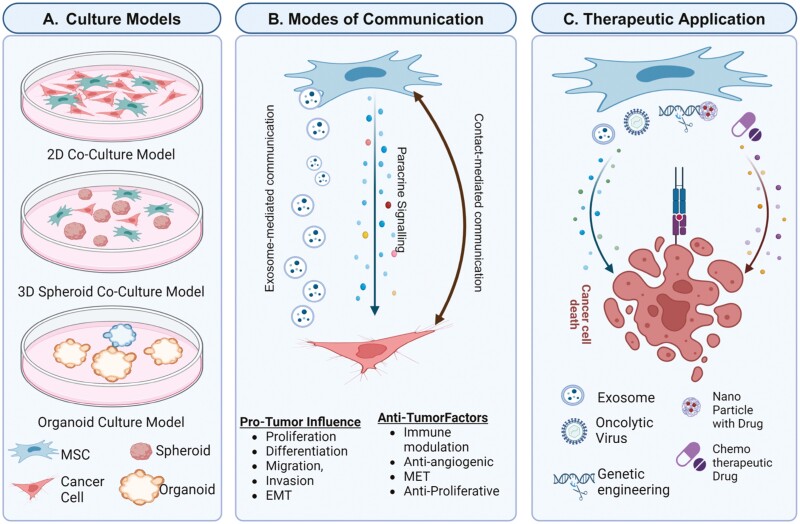

**Significance statement**

Mesenchymal stem cells play a significant role in interacting with tumor cells via paracrine signaling, affecting behaviors such as proliferation, migration, and epithelial-mesenchymal transition. In 3 dimensional culture systems, mimicking in vivo conditions, the relationship with tumor cells leads to understand the signaling and all the interactions with the tumor microenvironment. Moreover, the therapeutic potential of engineered mesenchymal stem cells emphases their role in targeted anti-cancer treatments.

## Introduction

The tumor microenvironment (TME) comprises a complex network of cells, mediators, and extracellular matrix (ECM) components that facilitate tumor growth and development. Mesenchymal stem cells (MSCs) in the TME contribute significantly to tumor progression via autocrine and paracrine signaling and cell-to-cell communication.^1^ While conventional 2D cell cultures have provided valuable insights into these interactions, they do not fully replicate the TME’s complexity. In contrast, 3D cell culture models offer improved cellular architecture, gene expression, metabolism, and signaling that more closely resemble in vivo conditions. Research has demonstrated that cancer cells behave differently in 3D coculture conditions than in 2D systems.^2^ Therefore, applying 3D culture systems to study MSC-tumor interactions holds significant promise for advancing cancer research and therapy development.

MSCs play a critical role in cancer development and therapy by mediating interactions with malignant cells through multiple signaling pathways, such as WNT/β-catenin, nuclear factor kappa B (NF-κB), and transforming growth factor beta (TGF-β). These pathways regulate various aspects of tumor progression, including epithelial-mesenchymal transition (EMT), metastasis, immune suppression, and vascular development.^1^ Understanding these multifaceted roles is crucial for developing MSC-based therapies, including their potential as delivery vehicles for chemotherapy drugs, oncolytic viruses, and immunotherapeutic cytokines. MSC-derived exosomes offer a promising cell-free therapeutic approach capable of transferring genetic materials to target cells.^3^

This review explores advancements in 3D culture models, intricate signaling pathways involved in MSC-cancer cell interactions, and the therapeutic potential of MSCs in cancer treatment.

## Culture models

The heterogeneity of the TME characterizes tumor properties, involving cell interactions, mediator secretion, and cell-to-cell communication, supporting tumor growth and progression through cellular and physical changes.^4^ The TME includes a variety of mesenchymal cell types that contribute to tumor development. MSCs are multipotent cells that reside in the niches of nearly all human tissues and organs, such as bone marrow, adipose tissue, the heart, the lung, and neonatal tissues such as the placenta, amniotic membranes, or the umbilical cord.^5,6^ MSCs share many characteristics, such as the expression of surface markers (CD73, CD90, and CD105), and can differentiate into adipogenic, chondrogenic, and osteogenic lineages.^7^ Applications of MSCs in cancer research and therapy are increasingly being explored due to their ability to modulate the TME and influence tumor growth and progression. Traditional 2D cultures are useful for identifying dysregulated signaling pathways, although primary MSCs and tumor cells cultured on flat surfaces cannot fully recapitulate the functional complexity of the TME.^4,8^ However, these 2D systems have provided valuable information on the intercellular communication between MSCs and tumor cells.

However, these 2D systems have provided valuable information on MSCs-tumor cell communication.

Cellular interactions are mediated via:

- *Paracrine signaling*: MSCs secrete various factors, including growth factors (eg, TGF-β, human growth factor), cytokines (eg, IL-6, IL-8), and chemokines (eg, CXCL12) that influence cell behaviors such as proliferation, migration, and invasion. Paracrine signaling promotes EMT by switching from E-cadherin to N-cadherin expression, tumor cell dissemination, and metastasis.^4^
- Cell organelle-mediated communication: MSC-derived exosomes deliver bioactive molecules, including miRNAs and proteins, to tumor cells, modulating their genetic expression and phenotype.^9^ Exosomes produced by MSCs have a dual role in chemoresistance and chemosensitivity, linked to specific ncRNAs and signaling pathway activation.^10^ Herst et al. also demonstrated that MSCs can donate mitochondria to respiration-deficient breast cancer cells (BCCs), enhancing their resistance to anticancer therapies by boosting ATP-binding cassette transporter activity.^11^ Del Vecchio et al. studied mitochondrial transfer (MT) by tunneling nanotubes (TNTs) from human adipose stem cells (hASCs) to BCCs in a 2D coculture system and observed that mitochondria derived from hASCs enhanced BCCs’ capacity to adapt to the extracellular environment, increasing their oxidative phosphorylation (OXPHOS) and mitochondrial respiration, and leading to a more chemoresistant phenotype.^12^

**Figure 1.**
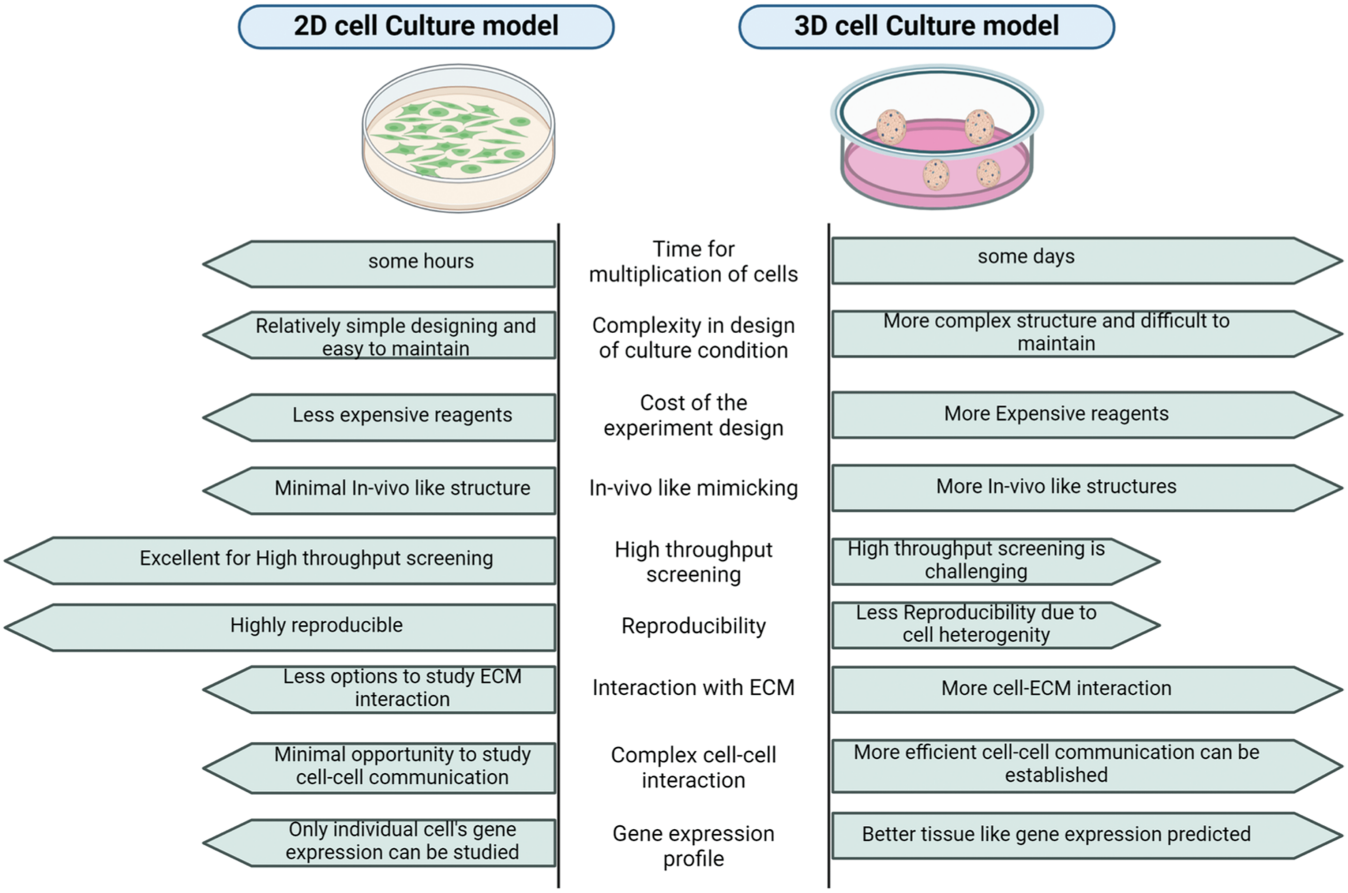
Comprehensive comparison between 2D and 3D cell culture.

The limitations of 2D cell culture prompted the development of 3D cell culture systems, which provide a more accurate representation of the TME. A comprehensive comparison between 2D and 3D culture has been provided in Figure 1. 3D coculture models featuring MSCs, and cancer cells have been established utilizing hanging drop assays or scaffolds sourced from natural materials (eg, collagen, hyaluronan, Matrigel, elastin, and chitosan) as well as those of synthetic origin (eg, polyethylene glycol, polyvinyl alcohol, and ceramics). Additionally, ECM derived from tumor biopsies is used to simulate the interstitial space and replicate microenvironmental conditions.^13,14^ The relatability of the used ECM biomaterial to that of the original TME predicts the efficiency of the 3D tumor model. Numerous studies have shown that 3D cell culture allows cancer cells to have a better cellular architecture, gene expression, metabolism, and signaling similar to in vivo conditions.^15^ For example, Del Vecchio et al. also studied MT in a patient-derived organoid model that gave a better physiological picture of the process. In their hybrid 2D/3D coculture model, the blockade of TNT scaffoldings did not significantly inhibit the MT, in contrast to the 2D coculture study, signifying that in this intricate spatial system, it takes place by additional cell-to-cell contact-driven mechanisms that need to be further explored.^12^ Moreover, co-culturing human pancreatic stellate cells (hPSC) with pancreatic cancer (PCa) cells in 3D models showed a loss of epithelial character and induction of the EMT in the Panc1 cell spheroid.^16^ Jafarpour et al. investigated MSC-derived exosome-based chemotherapy drug formulations on the 3D multicellular spheroid models of breast cancer cell lines BT-474 and MDA-MB-231, showing that exosomal formulations of Cisplatin and Paclitaxel induced higher toxicity and apoptosis in the 3D multicellular spheroids with lower drug concentrations.^17^ Mandel et al. used sodium alginate encapsulation to investigate the effect of Wharton’s jelly-derived mesenchymal stem cells (WJMSCs) on MDA-MB-231 and MCF-7 cell lines, finding significant differences in the paracrine profile and gene expression between cells seeded in an alginate 3D scaffold and a 2D monolayer culture.^18^

Crosstalk between MSCs and cancer cells in 3D cultures has been explored using single-cell RNA sequencing (scRNA-seq) that provides insights into the heterogeneity of MSCs and cancer cells within the TME, uncovering rare cell populations and transcriptional changes associated with crosstalk. Studies have identified subclones of MSCs, such as differentiation-resistant MSCs (DR-MSCs), which exhibit characteristics resembling cancer initiation cells.^19^ The multi-omics analysis of patient-derived colorectal cancer (CRC) organoids revealed multiple baseline protein and gene expression profiles that coincide with the therapy-resistant patient samples. This valuable data can enable the design of therapies that can target these specific genes and proteins.^20^

These findings highlight the superior potential and applicability of 3D cell culture in recreating the TME for both basic and translational research.

Advanced 3D models, such as bio fabrication and microfluidics, have been developed to better understand tumor processes such as apoptosis, proliferation, migration, invasion, stroma activation, cytokine secretion, oncogene activation, and stroma-mediated extravasation and intravasation, allowing for real-time evaluation. With 3D bioprinting technology, intricate 3D cellular structures can be created, enabling the deposition of cells in a predetermined spatial configuration. Horder et al. assessed 3D-printed human ASC spheroids in an extrusion-based bioprinting setup and evaluated the adipogenic differentiation within the printed spheroids into adipose microtissues.^21^ Dance et al. demonstrated the effect of ASCs on breast cancer cell invasiveness by fabrication of a microscale human breast tumor within a stroma containing adipocytes and ASCs.^22^ Saini et al. investigated the effect of tumor stem cell interaction and stromal stiffness in mediating tumor progression using a 3D micro-engineered organotypic tumor-stroma model.^23^ “Organ-on-a-chip” technologies, which integrate spheroids and organoids with microfluidics, have also emerged in cancer research. Tumor-on-a-chip can be used to simulate tumor growth and expansion, angiogenesis, and progression from early to advanced lesions involving EMT, tumor cell invasion, and metastasis.^24^ Zou et al. demonstrated the potential of PDOs in precision oncology, particularly in predicting the immunotherapy response of hepatocellular carcinoma (HCC). MSCs and peripheral blood mononuclear cells are cocultured to create an HCC organoid-on-a-chip, mimicking the original TME. This high-throughput drug screening platform allows for more precise prediction of patients’ responses to anti-PD-L1 drugs, thereby improving the quality of organoids.^25^

These approaches are more expensive and less practical but facilitate the identification of molecular mechanisms underlying diseases and novel biomarkers, which are particularly useful for drug screening.

## Signaling pathways mediated by MSC-cancer cell interaction

MSC-cancer cell interactions are complex, involving several paracrine signaling mechanisms that modulate pro- and anti-tumorigenic pathways.

- *PI3K/Akt/mTOR pathway*: This pathway is central in mediating MSCs’ impact on tumor progression, modulating the TME, promoting angiogenesis, inducing stemness, and supporting cancer cell survival. MSCs foster immune suppression, EMT, and metastasis in breast and gastric cancers by activating PI3K/AKT signaling pathways.^26^ In breast cancer, MSCs can stimulate mammosphere formation via epidermal growth factor secretion, with subsequent activation of the PI3K/Akt survival pathway. The bone marrow-derived MSC-conditioned medium significantly enhanced the progression of head and neck cancer by activating the PI3K/AKT signaling pathway.^27,28^
- *WNT/β-catenin pathway*: This pathway, involved in cell proliferation, differentiation, and migration, is significantly influenced by MSCs, which can either secrete WNT ligands or modulate the expression of WNT pathway components in cancer cells. Umbilical cord MSCs enhance metastasis and chemoresistance in cholangiocarcinoma, although MSC-secreted dickkopf-related protein 1 (DKK-1), a soluble WNT antagonist, can decrease the proliferation rate of leukemia cancer cells.^1^
- *Notch pathway*: MSCs, through the Notch pathway, enhance cancer cells’ immune suppression, EMT induction, and tumor angiogenesis. MSCs with miR-126 modification release proangiogenic factors and induce Notch ligands expression, enhancing angiogenesis.^29^ MSCs enhance the stemness and progression of PCa cells by activating the Notch1 pathway.^30^ MSC-induced EMT in pancreatic cancer cells is partially reduced by DAPT, highlighting Notch singling’s role in MSC-cancer interactions.^31^
- *Hypoxia-inducible factor (HIF) pathway*: Hypoxia, a condition characterized by low oxygen levels, is a common feature of the TME and influences function and cancer cell properties. MSCs express HIF-1α and HIF-2α under hypoxic conditions, crucial for self-renewal, and the generation of growth factors and cytokines. Hypoxia-adapted MSCs contribute to tissue repair and tumor progression by modulating angiogenesis, cell survival, differentiation, immunomodulation, and migration.^32-34^
- *NF-κB pathway*: This pathway’s activation in MSCs promotes pro-inflammatory cytokine production, impacting tumor cell behavior. MSCs influence the TME by modulating NF-κB activity in cancer and stromal cells.^35^
- *TGF-β pathway*: This is one of the most studied pathways due to its involvement in different types of cancers. Specifically, the mobilization of MSCs to tumor sites and the trans differentiation of MSCs into CAF-like cells are partially mediated by TGF-β1 derived from both cancer cells and tumor-educated-stromal cells.^6^ TGF-β, released by CAFs, influences EMT by modulating gene transcription in the nucleus through many distinct signaling pathways.^36^
- YAP/TAZ pathway: MSCs with activated Yes-associated protein (YAP) and transcriptional co-activator with PDZ-binding motif (TAZ) enhance cancer cell proliferation, migration, invasion, and metastasis.^37^ MSCs have been shown to release prostaglandin E2 (PGE2), which activates YAP in liver cells and promotes hepatocyte proliferation.^38^ The ability of hypoxia-conditioned MSCs to promote cancer progression was also observed in hepatocellular carcinoma through YAP-mediated lipogenesis reprogramming.^39^
- *RhoA pathway*: The RhoA signaling cascade is believed to play an essential role in the migration of MSC.^40^ The family of RhoGTPases directs a variety of cell responses including cell migration, adhesion, transcription, and growth.^41^ It has been observed that high directional migration of human MSCs permanently grown in hypoxia is associated with the enhanced activation of RhoA.^42^

## Therapeutic application

MSCs’ tumor-homing ability and potential to modulate the TME make them attractive candidates for targeted cancer therapies. MSCs can be engineered to deliver therapeutic agents, such as oncolytic viruses, genes, and cytokines, directly to tumors. In fact, given the presence of chemokines and cell-adhesion molecules on MSCs’ surface, these cells can migrate to the tumor site. Moreover, MSCs have low immunogenicity due to a lack of expression of MHC-II.^43^ Interestingly, some preclinical studies are based on genetically modified MSCs. MSCs can be engineered with genes encoding enzymes that can convert non-active pro-drugs into active molecules, such as MSCs engineered with the cytosine deaminase-uracil phosphoribosyl transferase (CD-UPRT) gene, encoding for the CD-UPRT enzyme that allows the transformation of the pro-drug 5-fluorocytosine (5-FC) into the anti-cancer drug 5-fluorouracil (5-FU). In this way, the pro-drug will be activated, especially at the tumor site.^44^

MSCs also exhibit some resistance to chemotherapeutic agents, which makes them a viable option for the delivery of anticancer agents. Usually, MSCs efficiently deliver chemotherapeutic drugs like Doxorubicin (DOX), Paclitaxel (PTX), and Gemcitabine (GCB) to tumor sites through passive diffusion and exosome delivery.^45-47^ Researchers have used nanoparticles to reduce drug efflux from MSCs for anticancer agents. Quantum dots, Arg-Gly-Asp motifs, and nanoparticles have been used to reduce drug efflux and control drug release from MSCs.^48,49^

Moreover, since cancer cells can escape the control mechanisms, such as apoptosis, another interesting use of genes for MSC engineering is the integration of genes involved in apoptosis pathways. In this regard, an MSC line has been generated by integrating the gene codifying for tumor necrosis factor-related apoptosis-induced ligand (TRAIL). TRAIL activates caspase-8, which is involved in the apoptotic extrinsic pathway and promotes cell death, especially in cancer cells rather than in normal cells.^50^

Oncolytic viruses are valuable in immuno-oncology, with some products in clinical development for adult cancers, and one approved for melanoma by the FDA and EMA. However, their anticancer clinical application is limited.^51,52^ The “Trojan horse” strategy uses MSCs as delivery vehicles to protect oncolytic viruses from the immune system and home them to tumor tissue. This strategy improves oncolytic viral therapy efficacy by augmenting the quantity of oncolytic virus administered, minimizing toxicities, and bypassing direct injection into tumors.^3^ In this context, a very motivating phase I/II clinical trial (NCT01844661) has been completed using Celyvir for treating metastatic and refractory solid tumors.^53^ Celyvir, an advanced therapy medicine, uses autologous BM-MSCs loaded with oAd for ICOVIR5 delivery. The study found Celyvir to induce beneficial antitumor effects with an excellent safety profile. Interestingly, they have reported complete healing in one pediatric patient 3 years after treatment with Celyvir.^54^ Some other clinical trials are also underway, and their outcomes are yet to be revealed (NCT05047276, NCT03896568, and NCT02068794).^55-57^

Locally produced immunotherapeutic cytokines can prolong release and amplify antitumor immune responses.^58^ Genetically engineered MSCs can overcome immunosuppressive conditions in tumor metastasis and control tumor progression. MSCs have been used as a vehicle for the selective delivery of a wide variety of cytokines, including IL-2, IL-12, and IFN-α in various cancer models.^59-61^

Mesenchymal stromal cell-derived extracellular vesicles (MSC-EVs) are crucial in tumor and stromal cell communication within the TME. EVs include exosomes, the smallest EV fraction from intracellular endosomes, and microvesicles generated by budding from the plasma membrane. They activate target cell surface receptors and deliver effectors, causing functional changes in recipient cells.^62^ Several studies suggest that the cell source may condition EV homing to specific sites and that their membrane can be engineered to increase tissue-specific targeting.^63^ These observations open new possibilities for future applications of MSC-EVs as cell-free therapeutic agents.^64,65^ MSC-derived exosomes may thus be used as delivery vehicles to transfer genetic materials, including mRNA, to recipient cells.^66-68^

## Conclusions and perspectives

The role of MSCs within the TME is multifaceted and crucial for tumor progression and response to therapies. Traditional 2D culture systems, while informative, fall short in replicating the TME’s complex interactions and architecture. Advancements in 3D culture models provide a more accurate representation of the TME, facilitating better understanding and development of MSC-based therapies.

From a therapeutic standpoint, MSCs hold great promise as delivery vehicles for anticancer agents, offering targeted and localized treatment options. Their low immunogenicity and tumor-homing capabilities make them highly suitable for delivering drugs, oncolytic viruses, and genetic therapies. Furthermore, the emergence of MSC-derived extracellular vesicles presents a novel, cell-free approach for targeted therapy, further highlighting the potential of MSCs in cancer treatment. In the meantime, novel strategies that target and reshape the TME to block tumor-promoting crosstalk and restore immune surveillance are immediately needed. A better understanding of the unique molecular mechanisms underlying these pro-tumorigenic events is crucial for improving current anti-cancer therapies.

To conclude, the 3D cell culture models developed using primary cells can provide better bio-mimicking models to study the intercellular communication between MSCs and tumor cells, and the knowledge gained from these models can help to design better therapeutic approaches using MSCs as delivery vehicles or as part of cell-based anticancer therapeutic regimes.

In any case, the potential risk of MSC-based therapies that promote cancer cell proliferation, metastasis, and therapeutic resistance must be carefully assessed, as they may have unintended consequences if not handled properly. Long-term safety studies are necessary to understand the implications of using MSCs and MSC-derived products in clinical settings. Ethical considerations such as ensuring informed consent for MSC source material and addressing regulatory challenges associated with genetically modified MSCs must also be taken into account.

## Data Availability

No new data were generated or analyzed in support of this research.
